# Co-ordinated regulation of gluconate catabolism and glucose uptake in *Corynebacterium glutamicum* by two functionally equivalent transcriptional regulators, GntR1 and GntR2

**DOI:** 10.1111/j.1365-2958.2007.06020.x

**Published:** 2007-11-06

**Authors:** Julia Frunzke, Verena Engels, Sonja Hasenbein, Cornelia Gätgens, Michael Bott

**Affiliations:** Institut für Biotechnologie 1, Forschungszentrum Jülich D-52425 Jülich, Germany.

## Abstract

*Corynebacterium glutamicum* is a Gram-positive soil bacterium that prefers the simultaneous catabolism of different carbon sources rather than their sequential utilization. This type of metabolism requires an adaptation of the utilization rates to the overall metabolic capacity. Here we show how two functionally redundant GntR-type transcriptional regulators, designated GntR1 and GntR2, co-ordinately regulate gluconate catabolism and glucose uptake. GntR1 and GntR2 strongly repress the genes encoding gluconate permease (*gntP*), gluconate kinase (*gntK*), and 6-phosphogluconate dehydrogenase (*gnd*) and weakly the pentose phosphate pathway genes organized in the *tkt-tal-zwf-opcA-devB* cluster. In contrast, *ptsG* encoding the EII^Glc^ permease of the glucose phosphotransferase system (PTS) is activated by GntR1 and GntR2. Gluconate and glucono-δ-lactone interfere with binding of GntR1 and GntR2 to their target promoters, leading to a derepression of the genes involved in gluconate catabolism and reduced *ptsG* expression. To our knowledge, this is the first example for gluconate-dependent transcriptional control of PTS genes. A mutant lacking both *gntR1* and *gntR2* shows a 60% lower glucose uptake rate and growth rate than the wild type when cultivated on glucose as sole carbon source. This growth defect can be complemented by plasmid-encoded GntR1 or GntR2.

## Introduction

*Corynebacterium glutamicum* is a predominantly aerobic, biotin-auxotrophic Gram-positive soil bacterium that was isolated in Japan owing to its ability to excrete l-glutamate under biotin-limiting growth conditions (Kinoshita *et al*., 1957). It is used today for the industrial production of more than two million tons of amino acids per year, mainly l-glutamate and l-lysine. Additionally, this species has become a model organism of the *Corynebacterineae*, a suborder of the *Actinomycetales* which also comprises the genus *Mycobacterium*. An overview on the current knowledge on *C. glutamicum* can be found in a recent monograph (Eggeling and Bott, 2005).

*Corynebacterium glutamicum* is able to grow on a variety of sugars, sugar alcohols and organic acids (e.g. acetate, lactate or citrate) as carbon and energy sources. The use of gluconate as an additional carbon source besides glucose was previously shown to have a positive effect on l-lysine production (Lee *et al*., 1998; Bianchi *et al*., 2001). In order to be metabolized, gluconate is first transported into the bacterial cytoplasm via a specific gluconate permease (GntP). Subsequently, it is phosphorylated to 6-phosphogluconate by gluconate kinase (GntK). In *C. glutamicum*, 6-phosphogluconate is further metabolized in the pentose phosphate pathway, as the alternative Entner–Doudoroff pathway is absent in this organism. Although in recent studies several transcriptional regulators involved in the regulation of central metabolic pathways in *C. glutamicum* were identified and characterized, knowledge about transcriptional regulation of genes involved in gluconate metabolism and pentose phosphate pathway is scarce (Gerstmeir *et al*., 2004; Kim *et al*., 2004; Krug *et al*., 2005; Cramer *et al*., 2006; Engels and Wendisch, 2007; Wennerhold *et al*., 2005; Bott, 2007).

In many bacteria genes involved in gluconate utilization are subject to negative control by GntR-like transcriptional regulators. In the case of GntR of *Bacillus subtilis* and *Escherichia coli*, it was shown that gluconate itself interferes with the binding of these regulators to their target promoters (Fujita and Fujita, 1987; Peekhaus and Conway, 1998). In several *Bacillus* species the genes encoding GntR, GntP, GntK, as well as a putative 6-phosphogluconate dehydrogenase (*gntZ*) are clustered in one operon. Expression of these genes is derepressed in the presence of gluconate and also subject to carbon catabolite repression by the catabolite control protein CcpA and the phosphocarrier protein HPr (Reizer *et al*., 1996). In *E. coli* the *gnt* genes are also repressed by the gluconate repressor GntR and activated by CRP (cAMP receptor protein) in complex with cAMP (Peekhaus and Conway, 1998). These data demonstrate that expression of the *gnt* genes is controlled in dependency of gluconate availability and the presence of a catabolite repressive carbohydrate-like glucose.

Recently, it was reported that the genes encoding gluconate permease and gluconate kinase (*gntP* and *gntK*) in *C. glutamicum* are also subject to carbon catabolite repression, presumably via the cAMP-dependent regulator GlxR which binds to the promoter regions of *gntP* and *gntK* (Letek *et al*., 2006). *C. glutamicum* GlxR contains a cAMP-binding motif and shows 27% sequence identity with the CRP protein of *E. coli*. GlxR was first identified as a repressor of *aceA* and *aceB* encoding the key enzymes of the glyoxylate cycle, isocitrate lyase and malate synthase respectively (Kim *et al*., 2004). Letek *et al*. (2006) reported that expression of *gntP* and *gntK* are not induced (or derepressed) by gluconate.

In this study, we have identified two paralogous GntR-type regulators in *C. glutamicum*, designated GntR1 and GntR2, which repress the expression of genes involved in gluconate metabolism (e.g. *gntK*, *gntP* and *gnd*) in the absence of gluconate. Surprisingly, these regulators function at the same time as activators of *ptsG* and *ptsS* encoding the permeases EII^Glc^ and EII^Suc^ of the PEP-dependent phosphotransferase system (PTS) for glucose and sucrose uptake in *C. glutamicum* (Lengeler *et al*., 1994; Kotrba *et al*., 2001; Parche *et al*., 2001; Moon *et al*., 2005). To our knowledge, this is the first example for a gluconate-dependent transcriptional control of PTS genes.

## Results

### Identification of putative gluconate-dependent transcriptional regulators in *C. glutamicum*

**Fig. 1:**
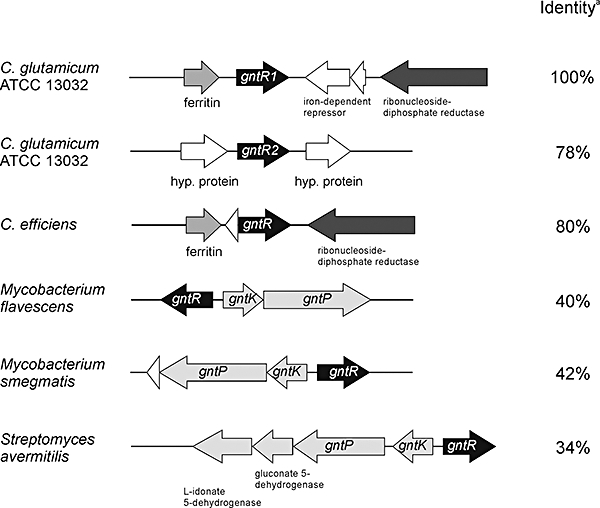
Genomic organization of GntR-type regulators with high sequence identity to GntR1. Genes for GntR-type regulators with high sequence identity to GntR1 from *C. glutamicum* are shown in black. In several *Mycobacterium* species and *Streptomyces avermitilis* genes encoding gluconate kinase (*gntK*) and gluconate permease (*gntP*) are located divergently to *gntR*. Data were taken from the bioinformatics software ERGO (Integrated Genomics). ^a^ Identity of the amino acid sequence to GntR1 (encoded by *cg2783*) of *C. glutamicum*.

In *C. glutamicum* genes involved in gluconate metabolism (*gntP*, *gntK*, *gnd*) are not clustered in an operon, like in *E. coli* or *B. subtilis*, but are scattered on the genome of this organism. In their close vicinity, no genes for transcriptional regulators belonging to the GntR family, which might act as gluconate-dependent regulators of these genes, could be detected. The genome of *C. glutamicum* ATCC 13032 contains 11 genes which encode GntR-type transcriptional regulators (Brune *et al*., 2005); two of them (*cg1935* and *cg2783*) show 78% sequence identity on the level of amino acid sequence and may have arisen by gene duplication. Interestingly, orthologs of *cg1935* and *cg2783* could also be found in *Mycobacterium flavescens* (Mflv_0501) and *Mycobacterium smegmatis* (MSMEG_0454) where they are located divergently to *gntK* and *gntP* (Fig. 1). This finding indicated a possible function of *cg1935* and *cg2783* in the regulation of gluconate metabolism in *C. glutamicum*. In *Corynebacterium efficiens*, an orthologous gene (CE2422) was located in a similar genomic context as *cg2783* in *C. glutamicum* (Fig. 1). Because of their proposed function in gluconate catabolism, the *C. glutamicum* genes were designated as *gntR1* (*cg2783*) and *gntR2* (*cg1935*). The sequence identity of GntR1 and GntR2 to GntR of *B. subtilis* and *E. coli*, which are known to control the expression of genes involved in gluconate metabolism, is below 30%.

GntR1 and GntR2 of *C. glutamicum* consist of an N-terminal GntR-type helix–turn–helix motif (PFAM: PF00392) responsible for DNA-binding and a C-terminal putative ligand-binding domain (PFAM: PF07729) typical for many GntR-type regulators. GntR-type regulators constitute to a large family of transcriptional regulators which typically share a highly conserved N-terminal DNA-binding motif, whereas the C-terminal parts show large divergence. Therefore, GntR members were classified into four subfamilies designated as FadR, HutC, MocR and YtrA (Rigali *et al*., 2002). Because of the presence of an FCD domain (FCD stands for FadR C-terminal domain) in GntR1 and GntR2 of *C. glutamicum*, these regulators most probably belong to the FadR family, which also includes GntR of *B. subtilis*. The coding region of *gntR2* (*cg1935*) lies within the prophage region CGP3 of the *C. glutamicum* genome (Kalinowski, 2005) which spans more than 180 kb covering approximately 200 coding regions for proteins most of which lack any significant similarities to known bacterial genes. In *C. glutamicum* strain R (Yukawa *et al*., 2007) and *C. efficiens* (Nishio *et al*., 2003), only orthologs of *gntR1* are present and located in the same genomic environment as *gntR1* of *C. glutamicum*.

### The genes *gntR1* and *gntR2* are functionally redundant

**Fig. 2:**
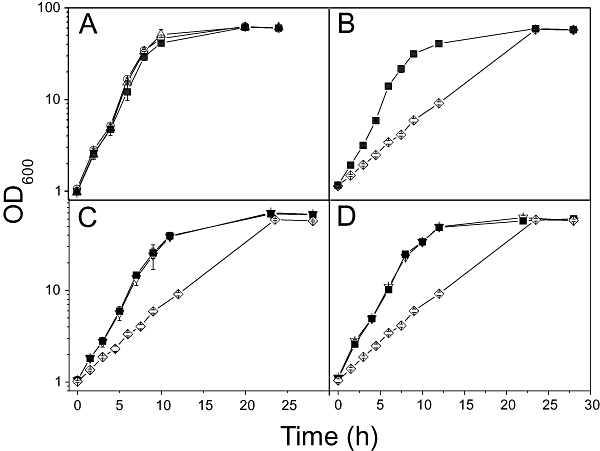
Growth of *C. glutamicum* wild type and different deletion mutants in CGXII minimal medium with 4% (w/v) glucose. In experiments C and D, the medium contained in addition 25 μg ml^−1^ kanaymycin. A. wild type (▪), Δ*gntR1* (Δ) and Δ*gntR2* (○). B. wild type (▪) and Δ*gntR1*Δ*gntR2* (◊). C. wild type/pAN6 (▪), Δ*gntR1*Δ*gntR2*/pAN6 (◊) and Δ*gntR1*Δ*gntR2*/pAN6-*gntR2* (∇). D. wild type/pAN6 (▪), Δ*gntR1*Δ*gntR2*/pAN6 (◊) and Δ*gntR1*Δ*gntR2*/pAN6-*gntR1* (∇).

In order to explore the regulatory function of GntR1 and GntR2 in *C. glutamicum* ATCC 13032, in-frame deletion mutants of the genes *cg2783* (Δ*gntR1*) and *cg1935* (Δ*gntR2*) as well as a double deletion mutant (Δ*gntR1*Δ*gntR2*) were constructed. Subsequently, growth of the different mutant strains was compared with that of the wild type using CGXII minimal medium containing either 4% (w/v) glucose or 2% (w/v) gluconate as carbon and energy source. When cultivated in minimal medium with 2% (w/v) gluconate, all four strains showed the same growth rate (0.46 ± 0.02 h^−1^) and the same final cell density (OD_600_ = 25 ± 1.5). In minimal medium with 4% (w/v) glucose, the mutant strains Δ*gntR1* and Δ*gntR2* displayed the same growth behaviour (μ = 0.41 ± 0.02 h^−1^, final OD_600_ = 60 ± 1.2) as the wild type (Fig. 2A). In contrast, the double mutant Δ*gntR1*Δ*gntR2* showed a strongly reduced growth rate of only 0.16 ± 0.01 h^−1^, but reached the same final cell density as the other strains after 24 h (Fig. 2B). As shown in Fig. 2C and D, the growth defect of mutant Δ*gntR1*Δ*gntR2* on glucose could be reversed by transformation with a plasmid carrying either the *gntR1* or the *gntR2* gene under control of the non-induced *tac* promoter. This result confirms that the simultaneous absence of GntR1 and GntR2 is responsible for the reduced growth rate in glucose minimal medium and indicates that GntR1 and GntR2 can replace each other. Complementation of the growth defect of strain Δ*gntR1*Δ*gntR2* on glucose was only possible when *gntR1* or *gntR2* were expressed at low levels owing to a basal activity of the *tac* promoter. Strong overexpression of either *gntR1* or *gntR2* in strain Δ*gntR1*Δ*gntR2* by addition of 1 mM isopropylthiogalactoside (IPTG) to the medium resulted in a growth defect in glucose and gluconate minimal medium, but not in acetate minimal medium (data not shown). Thus, high cellular levels of either GntR1 or GntR2 are inhibitory if glucose or gluconate are used as carbon source.

### Transcriptome analyses of the Δ*gntR1*, Δ*gntR2* and Δ*gntR1*Δ*gntR2* mutant strains

The growth experiments described above revealed that the single deletion mutants Δ*gntR1* and Δ*gntR2* grow like wild type under all tested conditions, whereas the Δ*gntR1*Δ*gntR2* deletion mutant shows a strongly reduced growth rate when cultivated on glucose, but not on gluconate. In order to elucidate the molecular basis of this phenotype, expression profiles of the different deletion mutants were compared with that of the *C. glutamicum* wild type using DNA microarray analysis. For this purpose, strains were cultivated in CGXII minimal medium with either 100 mM glucose or 100 mM gluconate. Additionally, expression profiles of wild type and the mutant Δ*gntR1*Δ*gntR2* were also compared after cultivation in CGXII minimal medium with 50 mM glucose and 50 mM gluconate. For each comparison, a set of two to three experiments starting from independent cultures was performed. RNA was isolated from cells harvested in the early exponential phase (OD_600_ 4–6) and always the expression levels of wild type and a deletion mutant were compared. No remarkable differences were observed between the expression levels of the single mutants Δ*gntR1* and Δ*gntR2* and the wild type, both for glucose- and gluconate-grown cells. A similar result was obtained in the comparison of wild type and the double mutant Δ*gntR1*Δ*gntR2* when the strains were cultivated either on gluconate alone or on glucose plus gluconate. In contrast, a variety of significant differences in gene expression was detected between wild type and strain Δ*gntR1*Δ*gntR2* when cells were cultivated with glucose as sole carbon source (Table 1).

**Table 1:** Genome-wide comparison of mRNA levels in *C. glutamicum* wild type with the mutant strains Δ*gntR1*, Δ*gntR1* or Δ*gntR1*Δ*gntR2* using DNA microarrays.

		mRNA ratio						
		
		Δ*gntR1*Δ*gntR2*/wild type			Δ*gntR1*/wild type		Δ*gntR2*/wild type	
				
Gene	Annotation	Glu	Glu + Gnt	Gnt	Glu	Gnt	Glu	Gnt
*cg1935*	Transcriptional regulator of GntR family, *gntR2*	< 0.01	0.05	< 0.01	2.09	1.00	0.05	< 0.01
*cg2783*	Transcriptional regulator of GntR family, *gntR1*	< 0.01	0.03	< 0.01	< 0.01	0.02	1.00	1.23
*cg2940*	Dipeptide transport ATP-binding protein, *dppF*	0.01	0.73	1.17	1.05	1.11	0.99	0.93
*cg0211*	Myo-inositol 2-dehydrogenase	0.02	1.12	2.66	1.18	1.32	1.13	0.93
*cg2691*	Hypothetical protein	0.02	0.92	0.77	0.99	1.02	0.90	0.84
*cg0751*	Hypothetical protein	0.03	1.08	3.23	1.10	1.09	0.87	0.85
*cg0082*	Chloride channel protein	0.03	1.36	1.69	1.04	1.06	1.10	0.95
*cg1493*	d-alanine–d-alanine ligase	0.03	1.28	0.90	1.25	1.19	1.00	1.16
*cg1369*	F_1_F_0_ ATP synthase ε subunit, *atpC*	0.03	1.05	5.36	1.44	1.14	0.84	0.97
*cg1537*	PTS system, glucose-specific IIABC component, *ptsG*	0.04	0.57	1.11	1.31	1.37	0.81	1.13
*cg0993*	Transcriptional regulator of ArsR family	0.05	0.74	7.14	0.93	0.98	0.93	0.95
*cg2725*	Transposase	0.08	0.69	0.45	0.37	0.65	1.34	2.83
*cg0658*	Hypothetical membrane spanning protein	0.08	0.89	0.91	1.13	1.06	0.99	0.90
*cg1488*	3-isopropylmalate dehydratase small subunit, *leuD*	0.10	1.23	1.09	1.27	1.15	0.98	0.98
*cg1451*	d-3-phosphoglycerate dehydrogenase, *serA*	0.12	1.10	0.86	1.00	1.09	0.67	1.14
*cg2125*	Uracil permease, *uraA*	0.14	1.11	1.22	0.86	1.10	1.03	0.98
*cg0564*	LSU ribosomal protein L1P	0.19	0.75	0.97	0.80	1.43	0.86	0.85
*cg0770*	ABC-type siderophore transport system, permease component	0.19	1.03	0.71	0.71	0.72	0.81	0.70
*cg0687*	O-sialoglycoprotein endopeptidase, *gcp*	0.19	1.24	0.67	0.80	1.13	0.88	1.03
*cg1002*	Hypothetical protein	0.19	0.91	0.99	0.81	0.86	0.93	1.22
*cg0286*	Transporter	0.21	0.67	1.20	0.90	1.16	0.99	1.10
*cg1487*	3-isopropylmalate dehydratase large subunit, *leuC*	0.21	1.76	1.31	1.05	1.24	1.34	0.92
*cg2399*	Glucose kinase, *glk*	0.22	0.93	0.82	0.66	1.09	0.91	0.81
*cg2925*	PTS system, sucrose-specific IIABC component, *ptsS*	0.26	1.05	1.37	1.17	1.47	1.14	1.28
*cg2178*	N utilization substance protein A	0.26	1.03	0.68	1.09	1.26	0.93	0.85
*cg3398*	Superfamily II DNA and RNA helicase	0.27	1.14	1.10	1.13	1.19	1.04	1.10
*cg1778*	Glucose 6-phosphate dehydrogenase, *zwf*	1.60	1.16	0.89	0.97	1.02	1.14	0.87
*cg1779*	Glucose 6-phosphate dehydrogenase, *opcA*	1.77	1.03	0.88	0.89	1.09	1.15	0.74
*cg1776*	Transaldolase, *tal*	2.01	1.21	0.81	1.06	1.02	1.12	0.97
*cg1780*	6-phosphogluconolactonase, *devB*	2.51	1.28	0.89	1.14	1.02	0.91	1.08
*cg1774*	Transketolase, *tkt*	2.87	1.13	0.83	1.07	1.09	1.03	1.12
*cg2836*	Succinyl-CoA synthetase α chain, *sucD*	4.48	0.93	0.52	1.41	0.75	0.81	0.97
*cg3399*	Hypothetical protein	4.50	1.85	0.76	0.83	1.01	1.22	0.97
*cg0143*	Mannitol 2-dehydrogenase	4.52	1.19	2.11	1.27	0.98	1.13	1.76
*cg0291*	Protocatechuate 3,4-dioxygenase β chain	4.64	0.75	0.72	0.91	1.14	1.19	1.11
*cg1454*	Taurine-binding protein	5.03	0.97	0.89	0.83	0.90	1.01	1.13
*cg1642*	Cytoplasmic siderophore-interacting protein	5.03	1.15	1.12	n.d.	1.11	1.15	1.55
*cg2616*	Vanillate O-demethylase oxygenase subunit	5.87	1.21	0.79	0.61	1.24	1.04	1.36
*cg0797*	Methylisocitrate lyase, *prpB1*	5.91	1.01	0.90	1.51	0.86	0.85	0.89
*cg0796*	2-methylcitrate dehydratase, *prpD1*	6.34	1.08	0.78	0.93	0.73	0.89	0.81
*cg0798*	2-methylcitrate synthase, *prpC1*	6.69	1.14	0.68	1.24	0.88	1.09	1.29
*cg0144*	Transporter	8.11	1.03	1.87	1.01	0.98	1.17	1.29
*cg1589*	Hypothetical protein	9.33	1.06	1.21	0.98	1.08	1.15	1.11
*cg1643*	6-phosphogluconate dehydrogenase, *gnd*	12.40	1.12	1.76	1.36	1.28	0.97	1.19
*cg3216*	Gluconate permease, *gntP*	25.08	0.79	0.83	1.38	0.91	1.33	1.07
*cg2810*	Na^+^/H^+^-dicarboxylate symport protein	65.46	1.04	1.29	1.20	1.04	1.01	2.41
*cg1255*	HNH endonuclease family protein	129.02	1.01	1.60	1.33	0.94	1.01	1.44
*cg2733*	HNH endonuclease family protein	155.10	1.05	1.46	1.01	0.95	1.05	11.53
*cg0385*	Periplasmic β-glucosidase/β-xylosidase, *bglS*	935.79	1.36	0.01	n.d.	1.27	1.77	21.08
*cg2732*	Gluconate kinase, *gntK*	2716.50	1.05	1.06	1.11	1.10	1.19	4.69

The mRNA ratios shown represent mean values from two or three independent microarray experiments starting from independent cultures (see *Experimental procedures*). In total, 17 microarray experiments were performed for the three comparisons Δ*gntR1* versus wild type, Δ*gntR1*Δ*gntR2* versus wild type and Δ*gntR2* versus wild type. The strains were cultivated in CGXII minimal medium with either 100 mM glucose (Glu), or 100 mM gluconate (Gnt), or 50 mM glucose and 50 mM gluconate (Glu + Gnt) and mRNA was prepared from cells in the exponential growth phase. The table includes those genes which showed a ≥ fourfold changed mRNA level (increased or decreased) in at least two of the three experiments comparing the double mutant Δ*gntR1*Δ*gntR2* versus wild type on glucose minimal medium and which had a *P*-value of ≤ 0.05. The genes are ordered according to the mRNA ratio of this comparison. In addition, the gene cluster encoding enzymes of the pentose phosphate pathway have been included, although their mRNA ratio was changed less than fourfold.

**Fig. 3:**
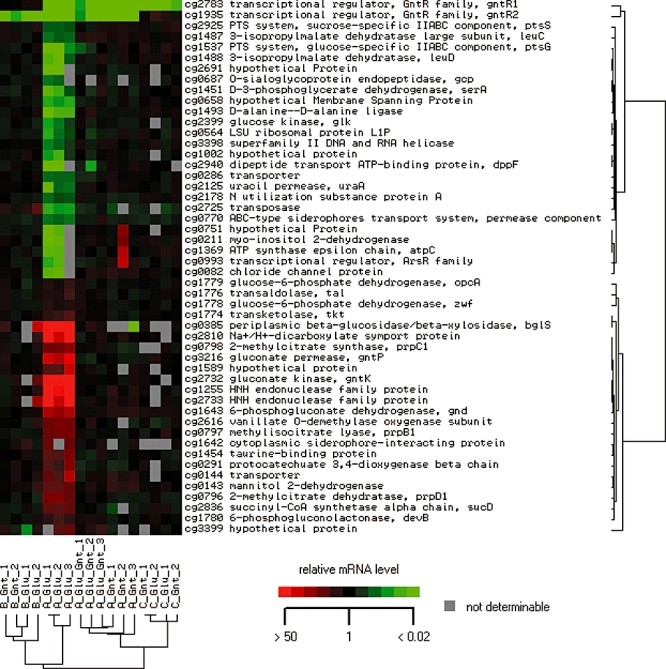
Hierarchical cluster analysis of gene expression changes in three series of DNA microarray experiments. The expression profiles of three different deletion mutants were compared with *C. glutamicum* wild type in totally 17 microarray experiments: (A) Δ*gntR1*Δ*gntR2* versus wild type; (B) Δ*gntR2* versus wild type; (C) Δ*gntR1* versus wild type. The strains were cultivated in CGXII minimal medium with either 100 mM glucose (Glu), or 100 mM gluconate (Gnt), or 50 mM glucose and 50 mM gluconate (Glu_Gnt). The cluster includes those genes which showed a ≥ fourfold changed mRNA level (increased or decreased) in at least two of the experiments A_Glu and had a *P*-value of ≤ 0.05. The relative mRNA level represents the ratio of mutant/wild type.

Figure 3 shows a hierarchical cluster of all genes which showed a ≥ fourfold altered mRNA level in the Δ*gntR1*Δ*gntR2* mutant cultivated on glucose. Under the chosen criteria, 26 genes showed a decreased and 19 genes an increased mRNA level in the Δ*gntR1*Δ*gntR2* mutant. Interestingly, one of the genes with the most significantly decreased mRNA level (factor 25) is *ptsG*, encoding the permease EII^Glc^ of the phospho*enol*pyruvate-dependent sugar PTS responsible for glucose uptake in *C. glutamicum* (Lee *et al*., 1994; Moon *et al*., 2005). Additionally, also the mRNA level of the *ptsS* gene encoding the EII^Suc^ permease involved in sucrose uptake was lower by a factor of four in the double mutant. On the other hand, the genes involved in gluconate uptake and metabolism showed a strongly increased mRNA level in the Δ*gntR1*Δ*gntR2* mutant (*gntP* 25-fold, *gntK* 2700-fold, *gnd* 12-fold). Besides the mRNA level of 6-phosphogluconate dehydrogenase (*gnd*), also the mRNA levels of other pentose phosphate pathway genes (*tkt-tal-zwf-opcA-devB*) showed a 1.6-fold to threefold increased mRNA level. Although the mRNA ratios of these genes did not exceed a factor of four, they were also included in the hierarchical cluster analysis shown in Fig. 3. The microarray data indicate an important function of GntR1 and GntR2 in gluconate metabolism and sugar uptake in *C. glutamicum*. Additionally, they support the assumption that GntR1 and GntR2 are able to complement each other, because no significant gene expression differences were detected between the single deletion mutants Δ*gntR1* and Δ*gntR2* and the wild type.

### Influence of GntR1 and GntR2 on the activity of gluconate kinase, 6-phosphogluconate dehydrogenase and glucose 6-phosphate dehydrogenase

The microarray data indicated that GntR1 and GntR2 act as repressors of the genes required for gluconate catabolism, i.e. *gntP*, *gntK*, *gnd* and other pentose phosphate pathway genes. To test whether the differences observed at the mRNA level are also present at the protein level, we determined the specific activities of gluconate kinase, 6-phosphogluconate dehydrogenase and glucose 6-phosphate dehydrogenase in cell-free extracts of wild type and the deletion mutant Δ*gntR1*Δ*gntR2*. For this purpose, the strains were cultivated in CGXII minimal medium with either 4% glucose or 2% gluconate or 1% of glucose and gluconate or 2% acetate and harvested in the early exponential phase (OD_600_ 4–6). As shown in Table 2, the activities of all three enzymes were significantly increased in the Δ*gntR1*Δ*gntR2* mutant when the cells were grown with glucose or acetate as carbon source. As expected from the transcriptome analysis, gluconate kinase showed the strongest increase, as its activity was below the detection limit (0.01 U mg^−1^) in wild type cells cultivated on glucose or acetate. The activities of 6-phosphogluconate dehydrogenase and glucose 6-phosphate dehydrogenase were increased ∼10-fold and approximately threefold, respectively, in the Δ*gntR1*Δ*gntR2* mutant grown on glucose, which is in very good agreement with the increase in the mRNA levels. When extracts of cells grown on gluconate or glucose plus gluconate were tested, the enzyme activities were also increased in strain Δ*gntR1*Δ*gntR2*, but to a much lower extent (≤ twofold). These data support the assumption that GntR1 and GntR2 act as gluconate-responsive repressors of genes involved in gluconate catabolism and the pentose phosphate pathway.

**Table 2:** Specific activity of gluconate kinase, 6-phosphogluconate dehydrogenase and glucose 6-phosphate dehydrogenase in *C. glutamicum* wild type and the Δ*gntR1*Δ*gntR2* mutant.

		Specific activity (U mg ^−1^)		
		
Strain	Carbon source	Gluconate kinase	6-Phosphogluconate DH	Glucose 6-phosphate DH
Wild type	Glucose	n.d.^a^	0.19 ± 0.02	0.15 ± 0.02
Δ*gntR1*Δ*gntR2*		2.37 ± 0.3	2.62 ± 0.18	0.49 ± 0.08
Wild type	Gluconate	0.92 ± 0.1	0.84 ± 0.01	0.35 ± 0.02
Δ*gntR1*Δ*gntR2*		1.50 ± 0.2	1.69 ± 0.03	0.40 ± 0.03
Wild type	Glucose + gluconate	0.60 ± 0.1	0.69 ± 0.03	0.11 ± 0.01
Δ*gntR1*Δ*gntR2*		1.20 ± 0.1	1.13 ± 0.10	0.16 ± 0.03
Wild type	Acetate	n.d.^a^	0.12 ± 0.01	0.03 ± 0.01
Δ*gntR1*Δ*gntR2*		1.27 ± 0.3	1.79 ± 0.08	0.16 ± 0.04

a n.d., not detectable (below 0.01 U mg^−1^).

The two strains were grown in CGXII minimal medium containing either 4% (w/v) glucose or 2% (w/v) gluconate or 1% glucose plus 1% gluconate or 2% acetate. Cells were harvested in the early exponential growth phase (OD_600_∼5). Enzyme activities were determined in cell-free extracts. The values for the specific activities represent means ± standard deviations from at least three independent cultivations.

The activity of all three enzymes measured in the derepressed background of a Δ*gntR1*Δ*gntR2* mutant was higher (∼25–60%) in glucose-grown cells compared with gluconate-grown cells. This difference could be due to a regulatory effect on the transcriptional level elicited by the influence of GntR1 and GntR2 on glucose uptake (see below).

### Activation of PTS-dependent sugar uptake via GntR1 and GntR2

In contrast to genes involved in gluconate metabolism and the pentose phosphate pathway, the genes *ptsG* and *ptsS* encoding the permeases EII^Glc^ and EII^Suc^ of the PTS system showed 25-fold or fourfold decreased mRNA levels in the Δ*gntR1*Δ*gntR2* mutant, respectively. In order to investigate a potential activation of *ptsG* expression by GntR1 and GntR2, reporter gene fusion analyses were performed. The plasmid pET2-*ptsG* containing the *ptsG* promoter region in front of a promoterless chloramphenicol acetyltransferase gene (Engels and Wendisch, 2007) was transferred into *C. glutamicum* wild type and the Δ*gntR1*Δ*gntR2* mutant. Subsequently, the two strains were grown in CGXII minimal medium with either a single carbon source (100 mM glucose or 100 mM gluconate) or mixed carbon sources (50 mM glucose + 50 mM gluconate). When cultivated on glucose, expression of the *ptsG–cat* fusion was ninefold lower in the Δ*gntR1*Δ*gntR2* mutant in comparison to the wild type (Table 3); showing that the reduced *ptsG* mRNA level observed in the microarray experiments is caused by reduced transcription. When cultivated on gluconate or glucose plus gluconate, the CAT activity of the mutant was only 1.5- to 1.8-fold lower than the activity of the wild type. These results can be explained by the assumption that *ptsG* expression is strongly activated by GntR1 and GntR2 in the absence of gluconate.

**Table 3:** Specific chloramphenicol acetyltransferase (CAT) activities of *C. glutamicum* wild type and the mutant Δ*gntR1*Δ*gntR2*, both carrying the promoter-probe plasmid pET2-*ptsG*.

	Specific CAT activities (U mg ^−1^)	
	
Carbon source(s)	*C. glutamicum* wild type/pET2-*ptsG*	*C. glutamicum*Δ*gntR1*Δ*gntR2*/pET2-*ptsG*
Glucose	1.84 ± 0.30	0.21 ± 0.05
Gluconate	0.61 ± 0.13	0.41 ± 0.05
Glucose + gluconate	0.77 ± 0.03	0.43 ± 0.05

The cells were grown in CGXII minimal medium with either 100 mM glucose or 100 mM gluconate or with 50 mM of both carbon sources. Enzyme activities were determined in cell-free extracts. The values for the specific activities represent means ± standard deviations from three independent cultivations.

### Binding of purified GntR1 and GntR2 to the promoter regions of putative target genes

**Fig. 4:**
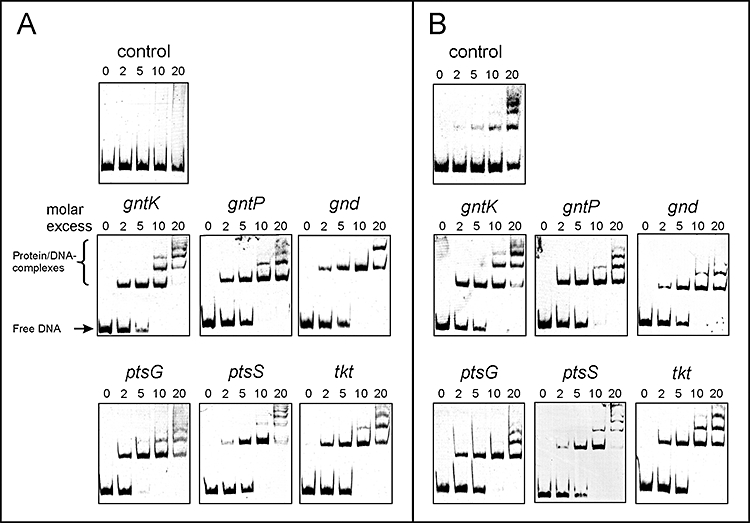
Binding of GntR1 (A) and GntR2 (B) to the promoter regions of the predicted target genes. DNA fragments (550 bp, 14 nM) covering the promoter regions of the putative target genes *gntP*, *gntK*, *gnd*, *ptsG*, *ptsS* and *tkt* were incubated for 20 min at room temperature either without protein or with a twofold, fivefold, 10-, or 20-fold molar excess of either purified GntR1 (A) or GntR2 protein (B). A DNA fragment containing the *acn* (aconitase) promoter region was used as a negative control. The samples were separated by native PAGE (10%) and stained with SybrGreen I.

The microarray experiments reported above identified *gntP*, *gntK*, *ptsG*, *ptsS* and the gene cluster *tkt-tal-zwf-opcA-devB* as putative target genes of GntR1 and GntR2. In order to test for a direct interaction of GntR1 and GntR2 with the promoter regions of these genes, the binding of the purified proteins was tested *in vitro*. For this purpose, GntR1 and GntR2 were overproduced in *E. coli* BL21(DE3)/pLysS and purified to homogeneity by means of an amino-terminal decahistidine tag (see *Experimental procedures*). The histidine tag does not interfere with the functionality of the proteins, as His-tagged GntR1 and GntR2 were able to complement the growth defect of the Δ*gntR1*Δ*gntR2* mutant on glucose (data not shown). In gel shift assays, DNA fragments covering the corresponding promoter regions were incubated with increasing concentrations of purified GntR1 or GntR2 and subsequently separated on a 10% native polyacrylamide gel. As shown in Fig. 4, all six promoter regions were shifted by GntR1 as well as by GntR2. A complete shift was observed at a fivefold to 10-fold molar excess of protein. Interestingly, at a 10- to 20-fold molar excess of protein, the formation of multiple GntR/DNA complexes was observed with all tested promoter regions. This observation could indicate the presence of several GntR1/2-binding motifs within the target promoter regions and/or the oligomerization of the protein once it is bound to DNA. Different DNA fragments covering for example the promoter regions of *acn* (aconitase) or *sdhCAB* (succinate dehydrogenase) served as negative controls and were incubated with the same protein concentrations as the putative target genes. The GntR2 protein also bound to these control DNA fragments, but with much lower affinity compared with the promoter regions of the identified target genes (Fig. 4B), indicating that this binding is unspecific.

**Fig. 5:**
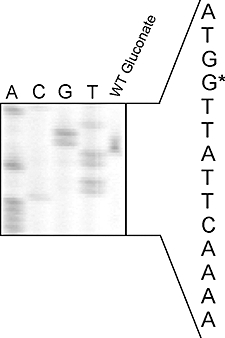
Identification of the transcriptional start site of the *gntK* gene by primer extension analysis using the oligonucleotide PE-gntK-1 (Table S1). Ten micrograms of total RNA isolated from *C. glutamicum* wild type grown on CGXII minimal medium with 100 mM gluconate was used as template. The transcriptional start site is indicated by an asterisk. The Sanger sequencing reactions (lanes A, C, G and T) were generated with a PCR product covering the corresponding DNA region as template and oligonucleotide PE-gntK-1.

In subsequent experiments the exact location of the binding sites of GntR1 and GntR2 was determined for four of the target genes (see below). In the case of *gntK*, the binding site was found to extend from position −45 to −59 with respect to the transcriptional start site reported by Letek *et al*. (2006), which is located 17 bp upstream of the ATG start codon. As the position of the binding site is unusual for a regulator acting as a repressor, we determined the transcriptional start site of *gntK* by primer extension analysis. A single primer extension product was detected using two independent oligonucleotides (PE-gntK-1 and PE-gntK-2, Table S1) and total RNA isolated from *C. glutamicum* wild type cultivated on minimal medium with 100 mM gluconate as carbon source. The transcriptional start site identified by these experiments is located 65 bp upstream of the start codon of *gntK* (Fig. 5). The extended ‘−10’ region derived from this start site (agagtTATGATag) shows a good agreement with the corresponding consensus sequence [tgngnTA(c/t)aaTgg] (Patek *et al*., 2003). No evidence for the previously reported transcriptional start site 17 bp upstream of the start codon was obtained in the primer extension experiments.

**Fig. 6:**
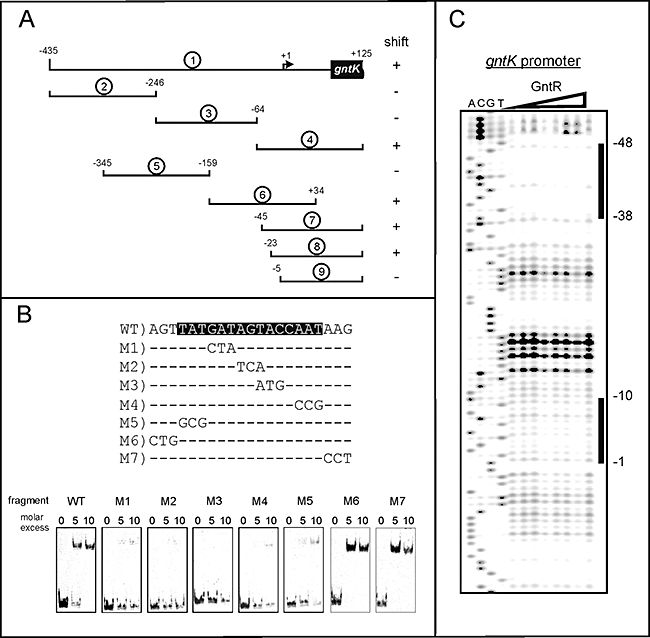
Identification of the GntR1/2 binding site in the promoter region of *gntK*. A. DNA fragments used to determine the location of the GntR1/2 binding site in the *gntK* promoter. The numbers indicate the position of the fragments relative to the transcription start site (+1) determined in this work (see Fig. 5). Oligonucleotides used for amplification by PCR are listed in Table S1. At the right, it is indicated whether the fragment, when tested in bandshift assays with purified GntR2, was shifted (+) or not (–). B. Mutational analysis of the putative GntR1/2 binding site (shaded in black) within the *gntK* promoter region. Mutations introduced are listed below the wild type sequence. Oligonucleotides used for amplification of the corresponding fragments are listed in Table S1. The fragments were incubated with purified GntR1 and the samples were separated on a 10% non-denaturating polyacrylamide gel and stained with SybrGreen I. C. DNase I footprinting analysis with GntR2 and the *gntK* promoter region. Two nM of IRD-800-labelled *gntK* template strand was incubated with increasing concentrations of GntR2 (0–2 μM). The first and the last lane were loaded with samples containing no protein. Regions protected from digestion by DNase I are indicated by the black bars. The DNA sequencing reactions were set up using the same IRD-800-labelled oligonucleotide as for generating labelled footprinting probes as well as suitable PCR template.

In order to identify the binding sites of GntR1 and GntR2 in the promoter region of *gntK*, the originally used DNA fragment was divided into several subfragments which were then also tested in gel shift assays with purified GntR1 and GntR2. As shown in Fig. 6A, GntR2 bound to fragments 4 and 6 which cover an overlapping region of approximately 100 bp. A further refinement using fragments 7–9 showed that an essential part of the GntR2 binding site is located between position −5 and −23 with respect to the transcription start site identified in this work. Further inspection of this region revealed a potential binding motif of GntR2 extending from position +4 to −11. Subsequently, the relevance of this motif was tested by mutational analysis. To this end, seven mutated DNA fragments were synthesized by PCR, each of which contained three nucleotide exchanges. All mutations within the postulated motif (fragments M1–M5) abolished binding of GntR2 (data not shown) and also of GntR1 (Fig. 6B) nearly completely, whereas the mutations outside the motif (fragments M6 and M7) had no effect on binding. These data confirm the relevance of the identified motif and show that GntR1 and GntR2 share the same binding site.

In an independent approach, the binding site of GntR1 and GntR2 within the *gntK* promoter was searched by DNase I footprinting. A protected region could be detected on the template strand extending from position −1 to −10 relative to the transcription start site, which completely overlaps with the binding motif previously identified by gel shift assays (Fig. 6C). This site was also confirmed by DNase I footprinting analysis with GntR1 and GntR2 and the non-template strand (data not shown). Interestingly, an additional protected region was present on the template strand between −38 and −48 (Fig. 6C). This indicates the existence of at least one additional GntR1/2 binding site, whose sequence shows no obvious similarity to those of the other identified GntR1/2 binding sites. Repression of *gntK* by GntR1 and GntR2 might involve formation of a DNA loop between the two binding sites.

**Fig. 7:**
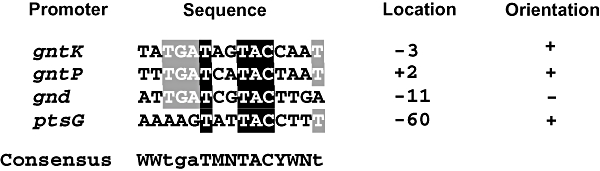
Experimentally identified GntR1/2 binding sites in the promoter regions of *gntK*, *gntP*, *gnd* and *ptsG*. The location of the central nucleotide of the 15 bp binding sites is indicated with respect to the transcriptional start site for *gntK, gntP* and *ptsG*, but with respect to the start codon for *gnd*. The orientation of the binding sites is indicated by plus and minus signs. The relevance of each binding site was confirmed by mutational analysis using gel shift assays with purified GntR1 and GntR2. Nucleotides shaded in black are conserved in all binding sites, those shaded in grey are identical in three of four binding sites.

Analysis of the promoter regions of *gntP*, *gnd* and *ptsG* by gel shift analyses with subfragments of the promoter regions also led to the identification of distinct sites involved in GntR1/2 binding (Fig. 7). The relevance of these sites was again confirmed by mutation studies which showed that an exchange of 3 bp within these sites prevented binding (data not shown). The binding sites were centred at position +2 with respect to the recently reported transcriptional start site of *gntP* (Letek *et al*., 2006) and at position −11 with respect to the start codon of *gnd*. In the case of *ptsG*, the binding site was centred at position −60 with respect to the transcriptional start site determined previously by primer extension experiments (Engels and Wendisch, 2007). These positions fit with a repressor function for *gntP* and *gnd* and an activator function for *ptsG* of GntR1/2. All GntR1/2 binding sites identified in this work are in reasonable agreement (1–2 mismatches) with a consensus operator site deduced for GntR-type regulators of the FadR subfamily (TNGTNNNACNA) (Rigali *et al*., 2002).

### Gluconate interferes with the binding of GntR1 and GntR2 to their target promoters

**Fig. 8:**
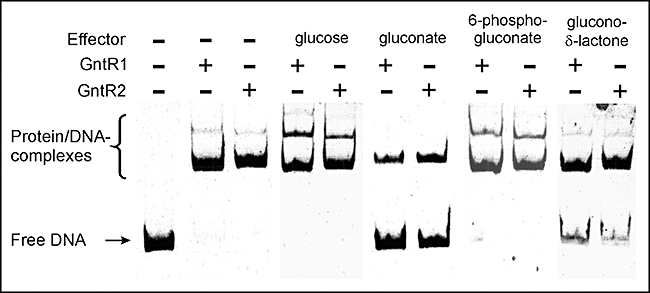
Search for putative effector molecules of GntR1 and GntR2. Various carbohydrates were tested for their influence on GntR1/2 binding to a DNA fragment containing the promoter region of *gntK*. Approximately 0.28 pmol of the 550 bp *gntK* fragment was incubated with either 2.8 pmol purified GntR1 or GntR2 protein in the presence of the following carbohydrates (50 mM each): glucose, gluconate, 6-phosphogluconate, glucono-δ-lactone. Not shown are the experiments with glucuronic acid, glucose 6-phosphate, fructose, sucrose, mannitol and sorbitol, which had no influence on DNA binding.

The transcriptome comparisons as well as the measurement of enzyme activities (gluconate kinase, 6-phosphogluconate dehydrogenase and glucose 6-phosphate dehydrogenase) indicated that the activity of GntR1 and GntR2 is dependent on the carbon source available. In order to identify putative effector molecules, the binding of GntR1 and GntR2 to the *gntK* promoter was assayed in the presence of glucose, gluconate, glucono-δ-lactone, 6-phosphogluconate, glucose 6-phosphate, fructose, sucrose, mannitol, sorbitol and glucuronate. For this purpose, purified GntR1 or GntR2 was incubated with the potential effector substances (50 mM) for 5–10 min before addition of a DNA fragment covering the *gntK* promoter and another 20 min of incubation. Subsequently, the samples were separated on a 10% native polyacrylamide gel. Of the 10 compounds tested only gluconate and, to a lower extent, glucono-δ-lactone inhibited binding of GntR1 and GntR2 to its target DNA (Fig. 8). In further studies it was shown that already a concentration of 1 mM gluconate led to a partial inhibition of binding. However, as even a concentration of 50 mM gluconate led only to a partial inhibition of binding, the possibility that a contaminating compound rather than gluconate or glucono-δ-lactone itself is responsible for the effect cannot be completely excluded. Similar results as described above for the *gntK* promoter were also obtained with the promoter regions of *gntP*, *gnd*, *tkt*, *ptsG* and *ptsS* (data not shown).

### Co-utilization of glucose and gluconate by *C. glutamicum*

**Fig. 9:**
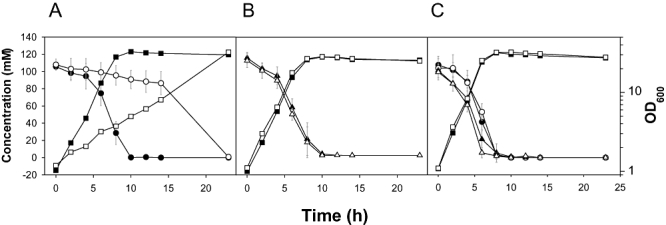
Growth (squares) and carbon source consumption of *C. glutamicum* wild type (filled symbols) and the mutant Δ*gntR1*Δ*gntR2* (open symbols). The two strains were cultivated in CGXII minimal medium containing as carbon source either 100 mM glucose (A), or 100 mM gluconate (B), or 50 mM glucose + 50 mM gluconate (C). The values are means obtained from three independent cultivations. Glucose and gluconate concentrations are indicated by circles and triangles, respectively.

It has previously been reported that *C. glutamicum*, like several other bacteria, is able to consume glucose and gluconate simultaneously (Lee *et al*., 1998). The results described above have uncovered that the genes involved in gluconate catabolism, including the pentose phosphate pathway, and the *ptsG* gene encoding the permease EII^Glc^ of the glucose PTS are co-ordinately regulated by GntR1 and GntR2. We therefore investigated whether the deletion of both transcriptional regulators has an effect on the co-consumption of glucose and gluconate. *C. glutamicum* wild type and the Δ*gntR1*Δ*gntR2* mutant were cultivated in CGXII minimal medium containing either 100 mM glucose, or 100 mM gluconate, or 50 mM glucose plus 50 mM gluconate and growth as well as glucose and gluconate uptake rates were monitored (Fig. 9). As described before, the Δ*gntR1*Δ*gntR2* mutant showed a drastically reduced growth rate when cultivated in minimal medium with 100 mM glucose (μ = 0.15 ± 0.01 h^−1^) in comparison to the wild type (μ = 0.43 ± 0.02 h^−1^). As expected from this observation, the glucose uptake rate of the Δ*gntR1*Δ*gntR2* mutant (33 nmol mg^−1^ min^−1^) was only one-third of that of the wild type (90 nmol mg^−1^ min^−1^) (Table 4). In contrast, cultivation on gluconate as carbon source resulted in almost identical growth rates of both strains (μ = 0.46 ± 0.02 h^−1^) and nearly identical gluconate uptake rates (99 nmol mg^−1^ min^−1^). The final cell density reached in gluconate medium (OD_600_ = 25.3 ± 0.5) was somewhat lower than the one reached in glucose medium (OD_600_ = 30.1 ± 1.1), which might be caused by an increased loss of substrate carbon as CO_2_ in the 6-phosphogluconate dehydrogenase reaction. In contrast to glucose, gluconate has to be metabolized completely via the oxidative pentose phospate pathway. Interestingly, when cells were cultivated with glucose plus gluconate, both *C. glutamicum* wild type and the Δ*gntR1*Δ*gntR2* mutant showed a significantly increased growth rate (μ = 0.52 ± 0.02 h^−1^). In this case, the final cell density (OD_600_ = 27.5 ± 0.3) was in between that obtained for glucose and gluconate as single carbon sources. Determination of the uptake rates confirmed that both strains consumed glucose and gluconate simultaneously. In the wild type, comparable uptake rates between 50 and 60 nmol mg^−1^ min^−1^ were determined (Table 4). Whereas the reduced glucose uptake in the wild type during cultivation in the presence of gluconate is presumably a consequence of the missing *ptsG* activation by GntR1 and GntR2, the reduced gluconate uptake in the presence of glucose might be caused by repression of *gntP* and *gntK* by the GlxR–cAMP complex, as suggested previously (Letek *et al*., 2006). In the Δ*gntR1*Δ*gntR2* mutant glucose uptake was slightly decreased compared with the wild type (52 versus 56 nmol mg^−1^ min^−1^), whereas gluconate uptake was slightly increased (65 versus 52 nmol mg^−1^ min^−1^). These minor differences might be explained by the assumption that in the wild type, but not in the Δ*gntR1*Δ*gntR2* mutant, there is some weak residual activation of *ptsG* and repression of *gntP*, *gntK* and *gnd* by GntR1 and GntR2 even in the presence of gluconate. Such a behaviour fits with the observation that even high gluconate concentrations did not completely prevent binding of GntR1/2 to its target promoters (see above). The finding that the glucose uptake rate of the Δ*gntR1*Δ*gntR2* mutant during growth on glucose plus gluconate was 50% higher than during growth on glucose alone indicates that gluconate has not only a negative effect on glucose uptake via GntR1/2, but also a positive effect via another transcriptional regulator or another regulatory mechanism.

**Table 4:** Carbon consumption rates of *C. glutamicum* wild type and the Δ*gntR1*Δ*gntR2* mutant during growth in CGXII minimal medium with either 100 mM glucose or gluconate or with 50 mM of both carbon sources.

	Carbon source consumption rates (nmol min^−1^ mg^−1^)		
	
Strain	Glucose	Gluconate	Glucose + gluconate
Wild type	90 ± 8	98 ± 9	56 ± 8; 52 ± 4
Δ*gntR1*Δ*gntR2*	33 ± 6	99 ± 8	52 ± 7; 65 ± 3

The represent means ± standard deviations for at least three independent cultivations.

## Discussion

In this study we have identified two functionally redundant GntR-type regulators in *C. glutamicum*, GntR1 and GntR2, which co-ordinately control gluconate catabolism and glucose uptake, presumably in dependency of the intracellular concentration of gluconate and glucono-δ-lactone. Whereas the negative control of genes involved in gluconate metabolism by GntR-type regulators has previously been demonstrated, e.g. in *E. coli* (Izu *et al*., 1997; Porco *et al*., 1997; Peekhaus and Conway, 1998) or *B. subtilis* (Miwa and Fujita, 1988; Fujita and Miwa, 1989; Reizer *et al*., 1991), the simultaneous positive control by these regulators of the *ptsG* gene encoding the key protein for glucose uptake via the PTS is a novel and surprising aspect. If the activation of *ptsG* expression is abolished by deletion of *gntR1* and *gntR2*, the growth rate and the glucose uptake rate of the corresponding strain in glucose minimal medium is reduced by about 60%. The question arises why this type of opposite co-regulation of glucose and gluconate metabolism has been established in *C. glutamicum*. One reason might be the fact that this species, in contrast to, e.g. *E. coli* or *B. subtilis*, usually prefers the simultaneous consumption of different carbon sources rather than their sequential utilization. Examples are the co-utilization of glucose with acetate (Wendisch *et al*., 2000), lactate (Stansen *et al*., 2005), propionate (Claes *et al*., 2002), fructose (Dominguez *et al*., 1997) or citrate (von der Osten *et al*., 1989). In the case of glucose-acetate co-metabolism it was shown that both the acetate consumption rate [270 nmol min^−1^ (mg protein)^−1^] and the glucose consumption rate [72 nmol min^−1^ (mg protein)^−1^] were twofold decreased compared with growth on acetate or glucose as sole carbon source, resulting in a comparable rate of total carbon uptake of about 1000 nmol C min^−1^ (mg protein)^−1^ under all three growth conditions (Wendisch *et al*., 2000). The carbon uptake rates [nmol C min^−1^ (mg protein)^−1^, based on the assumption that protein constitutes 50% of the cell dry weight] determined in this work for the wild type were in the same order of magnitude (Table 4): 1080 for growth on glucose, 1180 for growth on gluconate and 1290 for growth on glucose (670) plus gluconate (620). These two examples show that *C. glutamicum* is able to adjust the uptake rates for different carbon sources in such a way that they match its metabolic capacities. The co-metabolism of glucose and gluconate is advantageous for *C. glutamicum* as its growth rate (0.52 h^−1^) is increased by 20% compared with growth on glucose alone (0.43 h^−1^) and by 13% compared with growth on gluconate alone (0.46 h^−1^). Thus, activation of *ptsG* expression by GntR1 and GntR2 can be interpreted as one of the mechanisms that allow *C. glutamicum* the simultaneous consumption of carbon sources and thereby a maximization of its growth rate and a selective advantage in the competition with other microorganisms. Gluconate is likely to be a frequent substrate in nature, as (i) many bacteria, such as pseudomonads, acetic acid bacteria or enterobacteria (Neijssel *et al*., 1989; Anthony, 2004), possess membrane-bound glucose dehydrogenases that catalyse the extracytoplasmic oxidation of glucose to gluconic acid and (ii) a high number of bacteria possess gluconate permeases and are able to utilize gluconate either via the Entner–Doudoroff pathway or via the pentose phosphate pathway.

Besides its negative influence on *ptsG* expression mediated by GntR1 and GntR2, gluconate appears to have also a positive effect on *ptsG* expression: in the *ptsG–cat* fusion assays, expression of *ptsG* in the Δ*gntR1*Δ*gntR2* mutant was twofold higher on gluconate or glucose plus gluconate than on glucose alone (Table 3). Similarly, the glucose consumption rate of the double mutant was ∼60% higher during growth on glucose and gluconate than during growth on glucose alone (Table 4). These differences might be caused by the SugR protein, which was recently identified as a repressor of *ptsG* and other PTS genes during growth on gluconeogenic carbon sources (Engels and Wendisch, 2007). The activity of SugR is controlled by fructose 6-phosphate, which was shown to abolish binding of SugR to the *ptsG* promoter region *in vitro*. When gluconate is catabolized via the pentose phosphate pathway, it enters glycolysis at the level of fructose 6-phosphate and glyceraldehyde 3-phosphate. Therefore, it seems possible that the intracellular fructose 6-phosphate concentration is increased in the presence of gluconate and repression of *ptsG* by SugR is diminished. Analysis of *ptsG* expression in a Δ*gntR1*Δ*gntR2*Δ*sugR* triple mutant and measurement of the intracellular fructose 6-phosphate concentration might allow confirming or disproving this explanation.

The genomes of the closely related organisms *C. glutamicum* strain R (Yukawa *et al*., 2007) and *C. efficiens* contain just one *gntR* orthologous gene. Thus, the presence of *gntR2*, which most likely resulted from of a gene duplication event of *gntR1*, seems to be a characteristic of the *C. glutamicum* type strain ATCC 13032. As all results obtained in this work show that GntR1 and GntR2 can fully replace each other, the question arises why both *gntR* genes are retained in the chromosome. A convincing answer to this question is not yet available. The possibility exists that differences in the expression of the two genes or not yet uncovered individual functions of the regulators allow the cell a better adaptation to certain growth conditions.

In this work 10 direct target genes of GntR1 and GntR2 have been identified. Those involved in gluconate transport and metabolism (*gntP*, *gntK*, *gnd*, *tkt-tal-zwf-opcA-devB*) are repressed by GntR1 and GntR2, whereas *ptsG* and *ptsS* encoding the permeases EII^Glc^ and EII^Suc^ of the PTS system are activated. Activation of gene expression by GntR-type regulators has also been demonstrated for other members of this family, e.g. MatR, an activator of genes involved in malonate metabolism of *Rhizobium leguminosarum* (Rigali *et al*., 2002). Binding of GntR1 and GntR2 to all of its target promoters was inhibited by gluconate and glucono-δ-lactone (Fig. 8), which fits with their function in gluconate metabolism. The same metabolites were previously shown to interfere with binding of the *B. subtilis* GntR protein to its target promoters (Miwa and Fujita, 1988). Binding of *E. coli* GntR to the *gntT* promoter was likewise inhibited by gluconate, but at higher concentrations also by 6-phosphogluconate (Peekhaus and Conway, 1998). One millimolar and 20 mM gluconate were sufficient to completely inhibit binding of *E. coli* GntR and *B. subtilis* GntR to target promoters, respectively. In the case of *C. glutamicum* GntR1 and GntR2, only a partial inhibition of DNA binding was achieved with 50 mM gluconate, indicating a lower affinity for gluconate. Although the possibility exists that a contaminant present in the source of gluconate or glucono-δ-lactone could be responsible for inhibition of binding, this seems not very likely.

Besides being induced by gluconate, genes involved in the catabolism of this sugar acid are often subject to catabolite repression, e.g. in *E. coli* or *B. subtilis* (Reizer *et al*., 1996; Tong *et al*., 1996; Peekhaus and Conway, 1998; Titgemeyer and Hillen, 2002; Warner and Lolkema, 2003). Recently, it was reported that *gntK* and *gntP* of *C. glutamicum* are also subject to catabolite repression, mediated by the transcriptional regulator GlxR in complex with cAMP (Letek *et al*., 2006). Kim *et al*. (2004) reported that in *C. glutamicum* the cAMP concentration is 10-fold higher during growth on glucose than during growth on acetate, indicating that GlxR is active in the presence of glucose. Our finding that the gluconate consumption rate of *C. glutamicum* wild type is about twofold lower during growth on glucose plus gluconate compared with growth on gluconate alone (Table 4) could be due to catabolite repression of *gntP* and *gntK* by the GlxR–cAMP complex. A prerequisite for this explanation is that cells cultivated in the presence of glucose plus gluconate have a higher cAMP level than cells grown on gluconate alone.

In a previous study on gluconate metabolism in *C. glutamicum* it was reported that *gntP* and *gntK* are not induced by gluconate (Letek *et al*., 2006). Our results clearly show that *gntP* and *gntK* together with pentose phosphate pathway genes are induced by gluconate via GntR1 and GntR2. Simultaneously these regulators control glucose uptake by activation of *ptsG* expression in the absence of gluconate. In conclusion, these transcriptional regulators are important players in a complex regulatory network that controls uptake and metabolism of carbon sources in *C. glutamicum* in order to allow the most favourable combination of the available substrates.

## Experimental procedures

### Bacterial strains, media and growth conditions

All strains and plasmids used in this work are listed in Table 5. The *C. glutamicum* type strain ATCC 13032 (Kinoshita *et al*., 1957) was used as wild type. Strain Δ*gntR1* and strain Δ*gntR2* are derivatives containing an in-frame deletion of the genes *gntR1* (*cg2783*) and *gntR2* (*cg1935*), respectively. In strain Δ*gntR1*Δ*gntR2* both genes were deleted. For growth experiments, 5 ml of brain–heart infusion medium (Difco Laboratories, Detroit, USA) was inoculated with colonies from a fresh Luria–Bertani (LB) agar plate (Sambrook *et al*., 1989) and incubated for 6 h at 30°C and 170 r.p.m. After washing with 5 ml of 0.9% (w/v) NaCl, the cells of this first preculture were used to inoculate a 500 ml shake flask containing 50 ml of CGXII minimal medium (Keilhauer *et al*., 1993) with either glucose, or gluconate, or glucose plus gluconate in the indicated concentrations as carbon source(s). Additionally, the medium was supplemented with 30 mg l^−1^ 3,4-dihydroxybenzoate as iron chelator. This second preculture was cultivated overnight at 30°C and then used to inoculate the main culture to an optical density at OD_600_ of ∼1. The trace element solution was always added after autoclaving. For all cloning purposes, *E. coli* DH5α (Invitrogen, Karlsruhe, Germany) was used as host, for overproduction of the proteins Cg2783 (= GntR1) and Cg1935 (= GntR2) *E. coli* BL21(DE3)/pLysS. The *E. coli* strains were cultivated aerobically in LB medium at 37°C (strain DH5α) or at 30°C [strain BL21(DE3)/pLysS]. When appropriate, the media contained chloramphenicol [34 μg ml^−1^ for cultivation of *E. coli* BL21 (DE3)/pLysS], ampicillin (100 μg ml^−1^ for *E. coli*), or kanamycin (25 μg ml^−1^ for *C. glutamicum*, 50 μg ml^−1^ for *E. coli*).

**Table 5:** Bacterial strains and plasmids used in this study.

Strains or plasmids	Relevant characteristics	Source or reference
Strains		
*C. glutamicum*		
ATCC 13032	Biotin-auxotrophic wild type	Kinoshita *et al*. (1957)
Δ*gntR1*	In-frame deletion of the *gntR1* (*cg2783*) gene	This work
Δ*gntR2*	In-frame deletion of the *gntR2* (*cg1935*) gene	This work
Δ*gntR1*Δ*gntR2*	In-frame deletion of the genes *gntR1* and *gntR2*	This work
*E. coli*		
DH5α	*supE44*Δ*lacU169* (φ80*lacZ*DM15) *hsdR17 recA1 endA1 gyrA96 thi-1 relA1*	Invitrogen
BL21(DE3)/pLysS	F^–^*ompT hsdS*_B_(r_B_^–^m_B_^–^) *gal dcm* (DE3); contains plasmid pLysS (Cam^R.^)	Studier and Moffatt (1986)
Plasmids		
pK19*mobsacB*	Kan^R.^; vector for allelic exchange in *C. glutamicum*; (pK18 *oriV*_*E.c*._, *sacB*, *lacZ*α)	Schäfer *et al*. (1994)
pK19*mobsacB*-Δ*cg1935*	Kan^R.^; pK19*mobsacB* derivative containing a crossover PCR product covering the up- and downstream regions of *cg1935* (*gntR2*)	This work
pK19*mobsacB*-Δ*cg2783*	Kan^R.^; pK19*mobsacB* derivative containing a crossover PCR product covering the up- and downstream regions of *cg2783* (*gntR1*)	This work
pAN6	Kan^R.^; *C. glutamicum*/*E. coli* shuttle vector for regulated gene expression; derivative of pEKEx2 (*P*_tac_, *lacI*^q^, pBL1 *oriV*_*C.g.*_, pUC18 *oriV*_*E.c*._); for details see *Experimental procedures*	This work
pAN6-*gntR1*	Kan^R.^; pAN6 derivative containing the *gntR1* gene (*cg2783*) under control of the *tac* promoter	This work
pAN6-*gntR2*	Kan^R.^; pAN6 derivative containing the *gntR2* gene (*cg1935*) under control of the *tac* promoter	This work
pEKEx2	Kan^R.^; *C. glutamicum*/*E. coli* shuttle vector for regulated gene expression (*P*_tac_, *lacI*^Q^, pBL1 *oriV*_*C.g.*_, pUC18 *oriV*_*E.c.*_)	Eikmanns *et al*. (1991)
pEKEx2-*gntR1*-His	Kan^R.^; pEKEx2 derivative encoding GntR1 with an aminoterminal decahistidine tag	This work
pEKEx2-*gntR2*-His	Kan^R.^; pEKEx2 derivative encoding GntR2 with an aminoterminal decahistidine tag	This work
pET16b	Amp^R.^; vector for overexpression of genes in *E. coli*, adding a C-terminal hexahistidine affinity tag to the synthesized protein (pBR322 *oriV*_*E.c.*_, *P*_*T7*_, *lacI*)	Novagen
pET16b-*gntR1*	Kan^R.^; pET16b derivative for overproduction of GntR1 with an N-terminal decahistidine tag.	This work
pET16b-*gntR2*	Kan^R.^; pET16b derivative for over-production of GntR2 with an N-terminal decahistidine tag.	This work
pET2-*ptsG*	KanR; *C. glutamicum* promoter-probe plasmid with a 707 bp fragment covering the *C. glutamicum ptsG* promoter	Engels and Wendisch (2007)

### Recombinant DNA work

The enzymes for recombinant DNA work were obtained from Roche Diagnostics (Mannheim, Germany) or New England Biolabs (Frankfurt, Germany). The oligonucleotides used in this study are listed in Table S1 and were obtained from Operon (Cologne, Germany), except for the IRD800-labelled oligonucleotides, which were purchased from MWG Biotech (Ebersberg, Germany). Routine methods like PCR, restriction or ligation were carried out according to standard protocols (Sambrook *et al*., 1989). Chromosomal DNA from *C. glutamicum* was prepared as described (Eikmanns *et al*., 1994). Plasmids from *E. coli* were isolated with the QIAprep spin miniprep Kit (Qiagen, Hilden, Germany). *E. coli* was transformed by the RbCl method (Hanahan, 1985), *C. glutamicum* by electroporation (van der Rest *et al*., 1999). DNA sequencing was performed with a Genetic Analyzer 3100-Avant (Applied Biosystems, Darmstadt, Germany). Sequencing reactions were carried out with the BigDye Terminator v3.1 Cycle Sequencing Kit (Applied Biosystems, Darmstadt, Germany).

In-frame deletion mutants of *C. glutamicum* were constructed via a two-step homologous recombination procedure as described previously (Niebisch and Bott, 2001). The primers used for this purpose are listed in Table S1. The chromosomal deletions were confirmed by PCR with oligonucleotides annealing outside the deleted regions.

In order to complement the Δ*gntR1*Δ*gntR2* mutant, the *gntR1* (*cg2783*) and *gntR2* (*cg1935*) coding regions were amplified using oligonucleotides (2783NdeN, 2783Ex1, 1935NdeN and1935Ex1) introducing an NdeI restriction site that included the start codon and an NheI restriction site behind the stop codon. The resulting PCR products were cloned into the expression vector pAN6, resulting in plasmids pAN6-*gntR1* and pAN6-*gntR2*. These plasmids and as a control pAN6 were used to transform *C. glutamicum* wild type and the Δ*gntR1*Δ*gntR2* strain. The vector pAN6 is a derivative of pEKEx2 (Eikmanns *et al*., 1991) that contains a 56 bp insertion between the PstI and EcoRI restriction sites. This insertion harbours a ribosome binding site (GGAGATA) in an optimal distance to a unique NdeI cloning site. Downstream of the NdeI site, there is a unique NheI cloning site which is followed by a *Strep*Tag-II-coding sequence and a stop codon before the EcoRI site. For the construction of pAN6, the original NdeI restriction site of pEKEx2 was first removed by Klenow fill-in and religation and subsequently a DNA fragment of the sequence 5′-GACCTGCAGAAGGAGATATACATATGACCTGAGCTAGCTGGTCCCACCCACAGTTCGAGAAGTAAGAATTCGTC-3′ was cut with PstI and EcoRI and ligated with the modified pEKEx2 vector cut with the same enzymes.

For overproduction and purification of GntR1 and GntR2 with an N-terminal decahistidine tag, the corresponding coding regions were amplified using oligonucleotides that introduce an NdeI restriction site including the start codon and an XhoI restriction site after the stop codon. The purified PCR products were cloned into the expression vector pET16b (Novagen, Darmstadt, Germany), resulting in plasmids pET16b-*gntR1* and pET16b-*gntR2*. The GntR proteins encoded by these plasmids contain 21 additional amino acids (MGHHHHHHHHHHSSGHIEGRH) at the amino terminus. The PCR-derived portion of the constructed plasmids were analysed by DNA sequence analysis and found to contain no spurious mutations. For overproduction of the GntR proteins, the plasmids were transferred into *E. coli* BL21 (DE3)/pLysS.

### Global gene expression analysis

Preparation of RNA and synthesis of fluorescently labelled cDNA were carried out as described (Möker *et al*., 2004). Custom-made DNA microarrays for *C. glutamicum* ATCC 13032 printed with 70mer oligonucleotides were obtained from Operon (Cologne, Germany) and are based on the genome sequence entry NC_006958 (Kalinowski *et al*., 2003). Hybridization and stringent washing of the microarrays were performed according to the instructions of the supplier. Hybridization was carried out for 16–18 h at 42°C using a MAUI hybridization system (BioMicro Systems, Salt Lake City, USA). After washing the microarrays were dried by centrifugation (5 min, 1600 *g*) and fluorescence was determined at 532 nm (Cy3-dUTP) and 635 nm (Cy5-dUTP) with 10 μm resolution using an Axon GenePix 6000 laser scanner (Axon Instruments, Sunnyvale, USA). Quantitative image analysis was carried out using GenePix image analysis software and results were saved as GPR-file (GenePix Pro 6.0, Axon Instruments). For data normalization, GPR-files were processed using the BioConductor/R-packages limma and marray (http://www.bioconductor.org). Processed and normalized data as well as experimental details (MIAME, Brazma *et al*. 2001) were stored in the in-house microarray database for further analysis (Polen and Wendisch, 2004).

Using the DNA microarray technology, the genome-wide mRNA concentrations of *C. glutamicum* wild type were compared with those of the mutant strains Δ*gntR1*Δ*gntR2* (A), Δ*gntR2* (B), and Δ*gntR1* (C). The strains were cultivated in CGXII minimal medium with either 100 mM glucose, or 100 mM gluconate, or 50 mM glucose plus 50 mM gluconate (only for comparison A). RNA used for the synthesis of labelled cDNA was prepared from cells in the exponential growth phase. For each of the seven comparisons, two or three independent DNA microarray experiments were performed, each starting from an independent culture. To filter for differentially expressed genes and reliable signal detection in each of the seven comparisons, the following quality filter was applied: (i) flags ≤ 0 (GenePix Pro 6.0), (ii) signal/noise ≥ 3 for Cy5 (F635Median/B635Median, GenePix Pro 6.0) or Cy3 (F532Median/B532Median, GenePix Pro 6.0), (iii) ≥ fourfold change in the comparison Δ*gntR1*Δ*gntR2* mutant versus wild type in glucose minimal medium, and (iv) significant change (*P* < 0.05) in Student’s *t*-test (Excel, Microsoft).

### Primer extension analysis

For non-radioactive primer extension analysis of the *gntK* gene total RNA was isolated from exponentially growing *C. glutamicum* wild type cultivated in CGXII minimal medium with 100 mM gluconate as carbon source. Primer extension analysis with 10–13 μg of total RNA was performed using IRD800-labelled oligonucleotides (PE-gntK-1 and PE-gntK-2, Table S1) (MWG Biotech, Ebersberg, Germany) as described previously (Engels *et al*., 2004). The template for the DNA sequence analysis used to localize the 3′ end of the primer extension product was amplified in a standard PCR reaction using the unlabelled oligonucleotides gntK-seq-for and gntK-seq-rev (Table S1). The oligonucleotides PE-gntK-1 or PE-gntK-2 served as primers for the sequencing reactions.

### Measurement of enzyme activities

For the measurement of enzyme activities, cells of *C. glutamicum* wild type and the double deletion mutant Δ*gntR1*Δ*gntR2* were cultivated in CGXII minimal medium with either 4% (w/v) glucose or 2% (w/v) gluconate up to the exponential growth phase (OD_600_∼5). Then cells of 20 ml culture were harvested with ∼25 g of crushed ice (precooled to −20°C) by centrifugation at 4000 *g* for 5 min. The cell pellet was resuspended in 900 μl of Tris/HCl (50 mM, pH 7.5) and the cells were mechanically disrupted by 3 × 20 s bead beating with 1 g of zirconia-silica beads (diameter 0.1 mm; Roth, Karlsruhe, Germany) using a Silamat S5 (Vivadent, Ellwangen, Germany). After centrifugation (5 min, 18 320 *g*, 4°C), the supernatant was used immediately for the enzyme assay.

For the determination of glucose 6-phosphate dehydrogenase and 6-phosphogluconate dehydrogenase activity, the assay mixtures (1 ml total volume) contained 50 mM Tris/HCl pH 7.5, 10 mM MgCl_2_, 1 mM NADP^+^, 200 mM potassium glutamate and 3–20 μl cell-free extract (1–5 mg protein ml^−1^). The reaction was initiated by the addition of 4 mM glucose 6-phosphate or 1 mM 6-phosphogluconate, and the increase in absorption at 340 nm was monitored at 30°C using a Jasco V560 spectrophotometer (Jasco, Gross-Umstadt, Germany).

Gluconate kinase activity was determined in a coupled assay with 6-phosphogluconate dehydrogenase. The assay mixture (1 ml total volume) contained 50 mM Tris/HCl pH 8.0, 0.25 mM NADP^+^, 1 mM ATP, 1.2 U 6-phosphogluconate dehydrogenase, and 5–50 μl cell-free extract (1–5 mg protein ml^−1^). After preincubation for 5 min at 30°C, the reaction was started by the addition of 50 μl of a 200 mM gluconic acid solution (pH 6.8) and the increase in absorption at 340 nm was measured at 30°C.

### Chloramphenicol acetyltransferase assay

For analysing the expression of the *ptsG* gene, *C. glutamicum* wild type and the double mutant Δ*gntR1*Δ*gntR2* were transformed with plasmid pET2-*ptsG* (Engels and Wendisch, 2007), which is based on the corynebacterial promoter-probe vector pET2 (Vasicova *et al*., 1998) and contains the *ptsG* promoter region (−399 to +309) in front of a promoter-less *cat* (chloramphenicol acetyltransferase) gene. The promoter activity was tested by measuring chloramphenicol acetyltransferase activity in cell extracts. For this purpose, 5 ml of LB medium was inoculated with colonies from a fresh LB agar plate and incubated for 6 h at 30°C and 170 r.p.m. After washing the cells in CGXII medium without carbon source, the second preculture and subsequently the main culture (both 60 ml of CGXII minimal medium with 25 μg ml^−1^ kanamycin) were inoculated to an OD_600_ of 0.5. As carbon and energy source either 100 mM glucose, or 100 mM gluconate, or 50 mM glucose plus 50 mM gluconate was used. Precultures and main cultures were incubated at 30°C and 120 r.p.m. on a rotary shaker in 500 ml baffled shake flasks. The preparation of the crude extract and the measurement of its chloramphenicol acetyltransferase activity were performed as described by Engels and Wendisch (2007).

### Overproduction and purification of GntR1 and GntR2

The *C. glutamicum* proteins GntR1 and GntR2 containing 21 additional amino acids at the N-terminus (MGHHHHHHHHHHSSGHIEGRH) were overproduced in *E. coli* BL21(DE3)/pLysS using the expression plasmids pET16b-*gntR1* and pET16b-*gntR2*, respectively. Expression was induced at an *A*_600_ of 0.3 with 1 mM isopropyl β-d-thiogalactoside. Four hours after induction, cells were harvested by centrifugation and stored at −20°C. For cell extract preparation, thawed cells were washed once and resuspended in 10 ml of TNGI5 buffer (20 mM Tris/HCl pH 7.9, 300 mM NaCl, 5% (v/v) glycerol, and 5 mM imidazol). After the addition of 1 mM diisopropylfluorophosphate and 1 mM phenylmethylsulfonyl fluoride, the cell suspension was passed three times through a French pressure cell (SLM Aminco, Spectronic Instruments, Rochester, NY, USA) at 207 MPa. Intact cells and cell debris were removed by centrifugation (15 min, 5000 *g*, 4°C), and the cell-free extract was subjected to ultracentrifugation (1 h, 150 000 *g*, 4°C). GntR1 or GntR2 present in the supernatant of the ultracentrifugation step was purified by nickel chelate affinity chromatography using nickel-activated nitrilotriacetic acid-agarose (Novagen, Darmstadt, Germany). After washing the column with TNGI50 buffer (which contains 50 mM imidazol), specifically bound protein was eluted with TNGI100 buffer (which contains 100 mM imidazol). Fractions containing GntR1 or GntR2 were pooled, and the elution buffer was exchanged against TG buffer (30 mM Tris/HCl pH 7.5, 10% (v/v) glycerol).

### Gel shift assays

For testing the binding of GntR1 and GntR2 to putative target promoters, purified protein was mixed with DNA fragments (100–700 bp, final concentration 8–20 nM) in a total volume of 20 μl. The binding buffer contained 20 mM Tris/HCl pH 7.5, 50 mM KCl, 10 mM MgCl_2_, 5% (v/v) glycerol, and 0.5 mM EDTA. Approximately 13 nM promoter fragments of putative non-target genes of GntR1/2 (*acn*, *sucCD* and *sdh*) were used as negative controls. The reaction mixtures were incubated at room temperature for 20 min and then loaded onto a 10% native polyacrylamide gel. Electrophoresis was performed at room temperature and 170 V using 1× TBE (89 mM Tris base, 89 mM boric acid, 2 mM EDTA) as electrophoresis buffer. The gels were subsequently stained with SybrGreen I according to the instructions of the supplier (Sigma-Aldrich, Taufkirchen, Germany) and photographed. All PCR products used in the gel shift assays were purified with the PCR purification kit (Qiagen, Hilden, Germany) and eluted in EB buffer (10 mM Tris/HCl pH 8.5).

### DNase I footprinting

Labelled DNA fragments were obtained by amplification with 5′-IRD800-labelled oligonucleotides (MWG Biotech, Ebersberg, Germany). The *gntK* promoter region was amplified using the oligonucleotides gntK-2-for-M* and gntK-prom-rev-M (labelled template strand). Binding reactions, DNase I digestion and DNA precipitation were performed as described previously (Engels *et al*., 2004). A sample of 1.4 μl was then loaded onto a denaturating 4.6% (w/v) Long Ranger (Biozym, Hamburg, Germany) sequencing gel (separation length 61 cm) and separated in a Long Read IR DNA sequencer (Licor, Bad Homburg, Germany). The DNA sequencing reactions were set up using one of the IRD-800-labelled oligonucleotides and a suitable unlabelled PCR product of the promoter region as template.

### Determination of glucose and gluconate

To determine the concentration of glucose or gluconate in culture supernatants, 1 ml sample of the culture was centrifuged for 2 min at 16 060 *g* and aliquots of the supernatant were used directly for the assay or stored at −20°C. d-glucose and d-gluconate were quantified enzymatically using a d-glucose/d-fructose or a d-gluconic acid/glucono-δ-lactone Kit, respectively (R-Biopharm, Darmstadt, Germany), as described by the manufacturer. Concentrations were calculated based on calibration curves with standards of glucose or gluconate. Uptake rates (nmol min^−1^ (mg dry weight)^−1^) for glucose and gluconate (Table 4) were calculated according to the following equation:


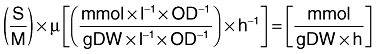


Where S is the slope of a plot of the substrate concentration in the medium versus the OD_600_ (mmol × l^−1^ × OD_600_^−1^), M the correlation between dry weight and OD (g dry weight × l^−1^ × OD^−1^) and μ the growth rate (h^−1^). An OD_600_ of 1 corresponds to 0.25 g dry weight l^−1^ (Kabus *et al*., 2007).

## Supplementary material

This material is available as part of the online article from:

http://www.blackwell-synergy.com/doi/abs/10.1111/j.1365-2958.2007.06020.x

(This link will take you to the article abstract).
