# Supplementary material for: Co-ordinated regulation of gluconate catabolism and glucose uptake in Corynebacterium glutamicum by two functionally equivalent transcriptional regulators, GntR1 and GntR2
Source: Mol Microbiol. 2007 Nov 6;67(2):305–22. doi: 10.1111/j.1365-2958.2007.06020.x (PMC2230225; doi:10.1111/j.1365-2958.2007.06020.x)
Supplement: Supplementary file 1 [file mmi0067-0305-SD1.pdf]

Table S1. Oligonucleotides used in this study.

Oligonucleotide	Sequence (5´→ 3´) and properties[a]
Oligonucleotides for *cg1935* and *cg2783* deletion and PCR analysis of the resulting mutants	
D1-1935	CAC AGC ACG TCT AGA CGG T (XbaI)
D2-1935	*CCC ATC CAC TAA ACT TAA ACA* AGG TAA AGC TGC GGA CAT AG
D3-1935	*TGT TTA AGT TTA GTG GAT GGG* AGG AGC ATT CTG AAT GA
D4-1935	AAG TGC CCC GGG TAT TCG G (XmaI)
D1935-fw	CACTGTTGAGGAAGGTAATGTCG
D1935-rv	ATGCTGCAGGTGTAGCAATGGC
D1-2783	TCG CTG CCA TCT AGA ACG C (XbaI)
D2-2783	*CCC ATC CAC TAA ACT TAA ACA* ACC TAA TGG ATT AGT CAT AGG A
D3-2783	*TGT TTA AGT TTA GTG GAT GGG* GCA GAA ACT GCG TCC CGA AA
D4-2783	TGT CTC CCC GGG TGT TGG A (XmaI)
D2783-fw	GTCACCTCCGCTATCGAGGC
D2783-rv	GTCTGAGAAGCTTGGATTAGGC
Oligonucleotides for expression of *gntR1* and *gntR2* in *C. glutamicum*	
1935-NdeN	TGAAAGAACATCATATGTCCGC (NdeI)
1935Ex1	GTAGTGGCTAGCTTAGTGGC (NheI)
2783-NdeN	TAAAGAGGCCATATGACCCC (NdeI)
2783Ex1	GACATTCCGCTAGCAGTGGC (NheI)
Oligonucleotides for overexpression of *gntR1* and *gntR2* in *E. coli*	
1935-NdeN	TGAAAGAACATCATATGTCCGC (NdeI)
1935-XhoC	GTAGTGACTCGAGTAGTGGC (XhoI)
2783-NdeN	TAAAGAGGCCATATGACCCC (NdeI)
2783-BamC	CCCTTGGATCCGCAAACGC (BamHI)
Oligonucleotides for generating PCR products used in gel shift assays	
gntK-prom-for	ATGGTGGCGTCATGCTCGGCCG
gntK-prom-rev	GGATTTGCCGCAGCCAGAAACGC
gntP-prom-for	GCTCTAGAGCCGCCCGAAACGCAG
gntP-prom-rev	CCGGCTGCAATGCCTAGCAGTGG
gnd-prom-for	CGTTGCTTCGGCCACGATGACAC
gnd-prom-rev	GGTTTGAGCCCATTACTGCTAGG
gntK-1-rev	GCGTGGGCCATCTCTTCGCGGAG
gntK-2-for	TTTGTATCAATGGAATCCGGGAC
gntK-2-rev	TGGTCACATCACGGGGACTGTATG
gntK-3-for	TACACACCACGGGGACTGTGGC
gntK-4-for	AGCGTGTGGAAAGAGTCATGTAC
gntK-4-rev	GATCAACGATTTGTTCGTATGTC
gntK-5-for	TAGTTGTGGAAACTTACGCCAGG
gntK-5-rev	TGCCGAATGCAGGAGGCTGCCAC
gntK-M2.0-for	AGAGTTATGATAGTACCAATAAGTTTTTGTGGCAG
gntK-M2.1	AGAGTTATCTAAGTACCAATAAGTTTTTGTGGCAG
gntK-M2.2	AGAGTTATGATTCAACCAATAAGTTTTTGTGGCAG
gntK-M2.3	AGAGTTATGATAGATGCAATAAGTTTTTGTGGCAG
gntK-M2.4	AGAGTTATGATAGTACCCCGAAGTTTTTGTGGCAG
gntK-M2.5	AGAGTGCGGATAGTACCAATAAGTTTTTGTGGCAG
gnd-2-for	CCCGGCCGGGCTCGATGGTGGTG
gnd-3-for	GGTGGCGGCAAGTTCTGCAAGCG
gnd-4-for	CCTCCTGTGACCTGGTAAAATCG
gnd-ohneM-1.0-for	CCCCCAAATGGTCACACCTTTTAGGC
gnd-ohneM-1.1-for	CCCCCAAATCCACACACCTTTTAGGCCGATTTTGC
gnd-M-1.2-for	CCCCCAAATGGTCTGTGCTTTTAGGCCGATTTTGC
gnd-M-1.3-for	CCCCGTTTTGGTCACACCTTTTAGGCCGATTTTGC
ptsG-M-1.0-for	ATTCAAAAGTATTACCTTTTTGGTTTGTC
ptsG-M-1.1-for	ATTCAAATCAATTACCTTTTTGGTTTGTCTGTATTCAG
ptsG-M-1.2-for	ATTCAAAAGTATATGGTTTTTGGTTTGTCTGTATTCAG
ptsG-M-1.3-for	ATTCAAAAGTATTACCGGGGTGTTTGTCTGTATTCAG
ptsG-M1.5	ATTCCCCAGTATTACCTTTTTGGTTTGTCTGTATTCAGCTTG
ptsG-M1.6	AGGAAAAAGTATTACCTTTTTGGTTTGTCTGTATTCAGCTTG
ptsG-M1.7	ATTCAAAAGTATTACCTTGGGGGTTTGTCTGTATTCAGCTTG

ptsG-M1.8	ATTCAAAAGTATTACCTTTTTTTGTTGTCTGTATTCAGCTTG
ptsG-prom-rev	GTTTCCCAGGCTCCCCCGCAATAG
Oligonucleotides for generating IRD800 labelled PCR products used in DNAse I footprints	
gntK-2-for-M*	TTTGTATCAATGGAATCCGGGAC
gntK-prom-rev-M*	GGATTTGCCGCAGCCAGAAACGC
Oligonucleotides used for primer extension analysis of *gntK* (labelled with IRD800)	
PE-gntK-1	GAAACGCCCATGACGACAATATG
PE-gntK-2	GAGCTCCGCTGCTAGGGCTTTAC
gntK-seq-for	AGCTGCTGCAGCTGAGCAATGTG
gntK-seq-rev	TTTCCTGGGGGTGAAGTTCGTCG

[a]In some cases oligonucleotides were designed to introduce recognition sites for restriction endonucleases (recognition sites underlined, restriction endonucleases indicated in parentheses) or complementary 21mer sequences for generating overlap-extension PCR products (printed in italics).
